# Drug Discovery Targeting the Disorder-To-Order Transition Regions through the Conformational Diversity Mimicking and Statistical Analysis

**DOI:** 10.3390/ijms21155248

**Published:** 2020-07-24

**Authors:** Insung Na, Sungwoo Choi, Seung Han Son, Vladimir N. Uversky, Chul Geun Kim

**Affiliations:** 1 Department of Life Science and Research Institute for Natural Sciences, Hanyang University, Seoul 04763, Korea; nainsung@hanyang.ac.kr (I.N.); cswya@naver.com (S.C.); imsangok12@naver.com (S.H.S.); 2 Department of Molecular Medicine, Morsani College of Medicine, University of South Florida, Tampa, FL 33612, USA; 3 Institute for Biological Instrumentation of the Russian Academy of Sciences, 142290 Pushchino, Russia; 4 CGK Biopharma Co. Ltd., 222 Wangshipri-ro, Sungdong-gu, Seoul 04763, Korea

**Keywords:** disorder-to-order transition, computer-aided drug discovery, protein–protein interaction inhibitor, conformational diversity mimicking, peptide docking

## Abstract

Intrinsically disordered proteins exist as highly dynamic conformational ensembles of diverse forms. However, the majority of virtual screening only focuses on proteins with defined structures. This means that computer-aided drug discovery is restricted. As a breakthrough, understanding the structural characteristics of intrinsically disordered proteins and its application can open the gate for unrestricted drug discovery. First, we segmented the target disorder-to-order transition region into a series of overlapping 20-amino-acid-long peptides. Folding prediction generated diverse conformations of these peptides. Next, we applied molecular docking, new evaluation score function, and statistical analysis. This approach successfully distinguished known compounds and their corresponding binding regions. Especially, Myc proto-oncogene protein (MYC) inhibitor 10058F4 was well distinguished from others of the chemical compound library. We also studied differences between the two Methyl-CpG-binding domain protein 2 (MBD2) inhibitors (ABA (2-amino-N-[[(3S)-2,3-dihydro-1,4-benzodioxin-3-yl]methyl]-acetamide) and APC ((R)-(3-(2-Amino-acetylamino)-pyrrolidine-1-carboxylic acid tert-butyl ester))). Both compounds bind MBD2 through electrostatic interaction behind its p66α-binding site. ABA is also able to bind p66α through electrostatic interaction behind its MBD2-binding site while APC-p66α binding was nonspecific. Therefore, structural heterogeneity mimicking of the disorder-to-order transition region at the peptide level and utilization of the new docking score function represent a useful approach that can efficiently discriminate compounds for expanded virtual screening toward intrinsically disordered proteins.

## 1. Introduction

Currently, computer-aided drug discovery represents a usual initiation of the drug development pathway. Among the computer-aided drug discovery approaches, computational hit screening is known as “virtual screening”, which uses ligand- or structure-based strategies and which is complementary to the traditional “high-throughput screening” methods utilized in wet-lab settings. Molecular docking is one of the representative techniques for virtual screening, and machine learning and deep learning techniques have become powerful tools nowadays [1]. Virtual screening depends on the knowledge of the structure of target proteins determined by X-ray crystallography, solution or solid-state NMR (Nuclear Magnetic Resonance), cryo-EM (Electron Microscopy), and sometimes homology modeling. This means that virtual screening is limited to the set of proteins with known structures, although disease does not always occur due to some problems pertaining to the ordered proteins. Furthermore, blocking such proteins is not always a perfect solution [2,3]. From this viewpoint, the major field of current computer-aided drug discovery process, virtual screening, is restricted to searches within a very limited space at the bottom of the funnel.

Intrinsically disordered proteins (IDPs) do not have defined structures; instead, they exist as highly dynamic conformational ensembles that rapidly change in response to subtle changes in the environment [4,5]. Not only can entire proteins be disordered but also many proteins represent hybrid entities containing ordered domains and intrinsically disordered protein regions (IDPRs) [6]. Some IDPRs function as binding sites for their corresponding partners through disorder-to-order transition, i.e., they utilize the binding-induced folding mechanism [7]. Such binding-induced disorder-to-order transition is characterized by structural transformation of a mostly unfolded unbound state into a specifically folded bound form. Such regions are commonly involved in molecular recognition, and therefore, they are known as molecular recognition features (MoRFs) [8,9]. Although such foldable intrinsically disordered recognition regions represent an attractive target for the discovery of protein–protein interaction inhibitors, for a long time, there were no drugs targeting disorder-based binding sites and there were reliable approaches for the utilization of these specific characteristics (binding-induced folding) in the drug discovery. However, the potential of this shift from ordered to disordered binding sites for the selection of promising drug targets was recently demonstrated by the traditional high-throughput screening-based discovery of the Myc proto-oncogene protein (MYC) inhibitors, including 10058F4 [10,11]. Furthermore, we recently elaborated an approach for the rational discovery of the inhibitors targeting such disorder-to-order transition regions and used this approach to find inhibitors targeting Methyl-CpG-binding domain protein 2 (MBD2) [12]. 

Earlier, inhibitors targeting disorder-to-order transition region of MYC involved in interaction with Protein Max (MAX) to form a tweezer-like coiled-coil dimer interacting with DNA were discovered using the yeast two-hybrid experiments [10]. Some compounds identified in this study were shown to possess antitumor efficacy *in vitro* and *in vivo* [10]. Later, two members of the library of these compounds, 10058F4 and 10074G5, targeting the MYC binding region were studied by a set of biophysical approaches [11]. The experiment employed mutagenesis and truncations of the MYC disorder-to-order transition region of MYC involved in interaction with MAX [13] and determined that 10058F4 binds to the region 402–412 of MYC whereas residues 363–381 were described as the 10074G5-binding region [11]. A recent comprehensive NMR analysis revealed the presence of subregions of a residual secondary structure within the MYC disorder-to-order transition region and structurally characterized regions affected by the 10058F4 binding [14]. This analysis revealed that the MYC 402–412 region serves as the 10058F4 target site and is embedded within the MYC disorder-to-order transition region responsible for MAX binding [14]. Curiously, according to our previous study, in the MYC per-residue intrinsic disorder profile, this 402–412 segment corresponds to a region with a high positive slope [12].

We also reported the discovery of two inhibitors targeting the disorder-to-order transition regions of MBD2 involved in an interaction with p66α [12]. We showed that the MBD2 segment that undergoes binding-induced disorder-to-order transition and forms coiled-coil dimer with its partner, p66α [15] (i.e., acts similarly to the MYC interacting with MAX [13]), is located within the region with a positive slope in the per-residue disorder profile of MBD2 [12]. By molecular docking utilized for virtual screening toward the coiled-coil disorder-to-order transition region of MBD2, we obtained two positive hits, ABA (2-amino-N-[[(3S)-2,3-dihydro-1,4-benzodioxin-3-yl]methyl]-acetamide) and APC (R)-(3-(2-Amino-acetylamino)-pyrrolidine-1-carboxylic acid tert-butyl ester). These compounds were shown to possess noticeable anti-metastasis activity due to the inhibition of the MBD2–p66α complex formation [12]. However, we did not analyze whether these two compounds can distinguish MBD2 and p66α (which has a disordered region that undergoes disorder-to-order transition while forming the coiled-coil heterodimer with MBD2).

Here, we developed a new method for escaping the bottom of the funnel that limits the structure-defined protein virtual screening preferentially to ordered proteins. We overcame this limit through segmentation of the target disorder-to-order transition region to a set of overlapping peptides, analysis of the structural preferences of these peptides, and elaboration of a new function for evaluation of the molecular docking score. We used this approach to study the binding mechanism of the MYC inhibitor (10058F4) toward its binding site in MYC. Besides, we also proved that the MBD2 inhibitors (ABA and APC) have different binding preferences toward MBD2 and p66α. We think that our novel method represents an important breakthrough that will expand the computer-aided drug discovery field, making possible the identification of inhibitors for proteins with unknown structures.

## 2. Results and Discussion

### 2.1. Computational Reevaluation of the 10058F4 Binding to the MYC Disorder-To-Order Transition Region

Earlier, we discovered the anti-metastasis activities of two compounds, ABA and APC (see the Section 3 Materials and Methods) by targeting MBD2 in molecular docking environment [12]. In the field of rational drug design, it is a commonly accepted practice to apply molecular docking toward target protein structures determined by X-ray crystallography, NMR, and cryo-EM. However, application of this powerful approach to intrinsically disordered proteins (IDPs) or intrinsically disordered protein regions (IDPRs) is challenging. In our previous study, we used a modified rational drug design approach for the discovery of drug leads based on molecular docking and molecular dynamics (MD) simulations of the IDPRs of target proteins capable of the disorder-to-order transitions (DOTs) [12]. The process started with the analysis of the intrinsic disorder predisposition of a drug target protein of interest followed by the prediction of the presence of potential disorder-based binding regions that can undergo DOTs. In parallel, a Protein Data Bank (PDB) search was conducted to find potential drug-target sites (DOT-based protein–protein interaction regions), and the corresponding structures were retrieved and used for molecular docking. This was combined with the evaluation of the off-target probabilities for the selection of lead compounds from the molecular-docked hit compounds. Finally, MD simulations [12] were used to evaluate the mode and efficiency of the prospected candidate compound binding. Although this approach targeted IDPRs (more precisely IDPRs capable of DOTs), by virtue of utilizing known structure of a target region in its bound state as a starting point for drug discovery, it used logistics of a conventional rational drug design.

In this study, we further developed this approach and moved it closer to the real-life settings by mimicking disorder-to-order transitions. Instead of considering a bound form of a long IDPR that underwent binding-induced folding and gained a specific structure, we focused on the intrinsic disorder nature of this region. To this end, we split each long IDPR of interest to a set of overlapping short peptides, used folding prediction algorithm to generate diverse conformational forms of these peptides, and then subjected these diverse conformations of model peptides to the molecular docking.

**Figure 1:**
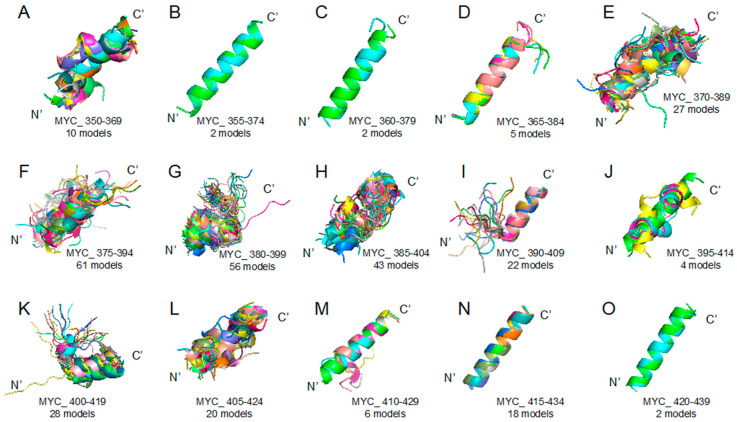
Myc proto-oncogene protein (MYC) 350–439 region segmented peptides: PEPFOLD ver 3.5 prediction result (see the Section 3 Materials and Methods) of peptide structures based on corresponding sequences (please look at the Table 1). (**A**) MYC_350–369 (10 models), (**B**) MYC_355–374 (2 models), (**C**) MYC_360–379 (2 models), (**D**) MYC_365–384 (5 models), (**E**) MYC_370–389 (27 models), (**F**) MYC_375–394 (61 models), (**G**) MYC_380–399 (56 models), (**H**) MYC_385–404 (43 models), (**I**) MYC_390–409 (22 models), (**J**) MYC_395–414 (4 models), (**K**) MYC_400–419 (28 models), (**L**) MYC_405–424 (20 models), (**M**) MYC_410–429 (6 models), (**N**) MYC_415–434 (18 models), (**O**) MYC_420–439 (2 models).

In more detail, the target regions used in this study were determined based on the structure for the MYC–MAX (PDB ID 1NKP) [13] complex by the X-ray crystallography. We considered the target DOT of MYC (residues 350–439) and segmented it into the sets of the overlapping 20-amino-acid-long sequences (Table 1). Next, each of the peptides was subjected to structure prediction using the PEPFOLD ver 3.5 web server (https://mobyle.rpbs.univ-paris-diderot.fr/cgi-bin/portal.py#forms::PEP-FOLD3) to predict its potential structures [16]. This stage generated a multitude of probable structures, which are exemplified by a set of conformations predicted for the 15 peptides overlapping derived from the MYC 350–439 region. Figure 1 clearly shows that different peptides possess very different folding potentials.

**Figure 2:**
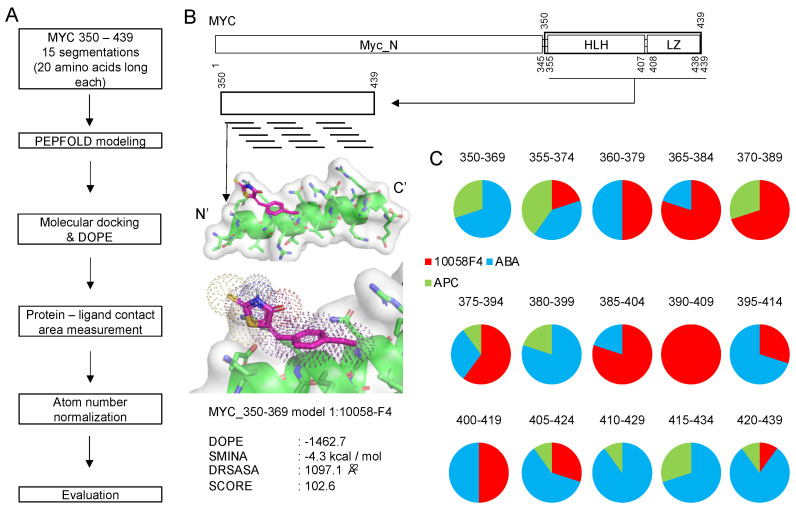
Peptide molecular docking procedure and its application to 10058F4 and other disorder-to-order transition region inhibitors (ABA and APC targeting MYC 350–439 region 15 peptide groups (Figure 1). (**A**) The procedure of target region segmentation and molecular docking toward generated peptides. (**B**) A workflow diagram following the procedure described in plot A. (**C**) Application of the method in the analysis of 10058F4, ABA, and APC interactions with 15 peptide groups derived from the MYC 350–439 region and pie charts showing distribution of the 10 best docking results for each peptide group.

Importantly, we designed a new evaluation score function focusing on the disorder-to-order transition region as described in the Section 3 Materials and Methods. The procedure of using such a segmented peptide for docking is summarized in Figure 2A,B.

To validate the applicability of this new approach for accurate discovery of small molecules inhibiting DOT-based protein–protein interactions, we aimed to distinguish a MYC disorder-to-order transition coiled-coil region inhibitor (10058F4) from other inhibitors (ABA and APC). Recent an NMR study described secondary structure regions in MYC and annotated structural changes induced by 10058F4 in this protein [14]. We expected that our approach would show results that would be similar or comparable with the outputs of this recent NMR study, especially for the higher rank docking results. After molecular docking of 10058F4 to 15 peptide groups, we discovered that the best 10 molecular docking outputs (see the Section 3 Materials and Methods) from each of the 15 groups showed two distinguishable interaction parts separated by the 380–399 region (Figure 2C). These observations follow the results of a recent NMR study [14].

According to the previous research on MYC inhibitors, the MYC 363–381 region is involved in 10074G5 binding and the 402–412 region plays a role in 10058F4 binding [11]. Our peptide docking study revealed that the 10 best positions from the MYC 390–409 region interact only with 10058F4. Because our peptide segmentation slides 5 amino acids, surrounding peptide docking results also showed higher 10058F4 occupancy in the 10 best docking results (Figure 2C). This region overlaps with the already known 10058F4 binding site (residues 402–412). Especially Y402 was discovered as the major binding residue from the previous circular dichroism and NMR experiment, which revealed Y402 activity in the MYC 402–412 under 10058F4 treatment [11], and two molecular simulation studies confirmed the role of Y402 in binding [17,18]. These previous studies support our discovery of the regions of major occupancy of 10058F4 in MYC 385–424 region peptides.

In summary, molecular docking traditionally relied on already determined target protein structures. As a result, it was hard to apply the technique toward the disorder-to-order transition region in noncomplex forms. Another problem is that intact protein corresponding binding site in MYC is so long to study disorder-to-order transition. To overcome the problems and to follow disorder-to-order transition definition, it was necessary to predict possible conformations in shorter length peptides derived from the binding site. Thus, we utilized web algorithm to predict diverse forms of binding site originated peptides. Molecular docking was applied toward peptides in diverse conformation. Finally, we proved well-known MYC inhibitor (10058F4) bindings toward corresponding disorder-to-order transition region.

### 2.2. MYC and MDM2 Inhibitors Docking Evaluation Shows Each Compound Binds to Peptide Corresponding Regions, and 10058F4 Interacts with MYC Y402

In the previous study, we focused on the similarity of the intrinsic disorder score pattern (positive slope) within the disorder-to-order transition coiled-coil regions of MYC and MBD2 [12]. Interestingly, the higher occupancy rank of 10058F4 was discovered for the MYC 360–394 region (Figure 2C), which is known as the 10074G5 binding site [11]. The higher occupancy of 10058F4 among the MYC-derived peptides could be due to their high protein-ligand activity, as a recent NMR discovered [14]. To compare with its original binding partner, we tested the interaction of 10074G5 with these peptides. Furthermore, we further tested the relevance using another well-known protein intrinsic disorder advantage for drug discovery, RG7112, an MDM2 inhibitor [19,20]. We determined the RG7112 binding site according to the structure of the humanized *Xenopus* MDM2, which showed that the corresponding human MDM2 binding site is located at the 50–99 region [21].

We applied the same molecular docking environment to 10074G5 and RG7112 (see the Section 3 Materials and Methods) with corresponding targets (10074G5–MYC and RG7112–MDM2). From each peptide docking result, we selected the 10 best positions (samples) and obtained T scores for them. We considered each compound docking result as a population for statistic calculation.

**Figure 3:**
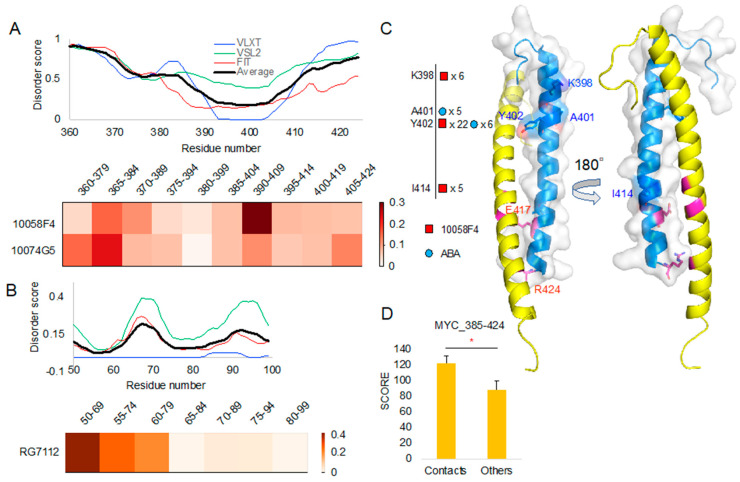
A protein intrinsic disorder score plot of peptide docking region and the corresponding region docking T score heatmap (normalized T scores): (**A**) MYC protein intrinsic disorder score and corresponding region T score heatmap of 10058F4 and 10074G5. (**B**) MDM2 protein intrinsic disorder score and corresponding region T score heatmap of RG7112. (**C**) MYC contact residues shown on MYC–Protein max (MAX) complex structure (PDB ID 1NKP) [13]. (**D**) *T*-test on the new docking evaluation score (*y*-axis) between contact mapping cases and others. * *p*-value < 0.001.

Importantly, our segmented peptide docking and evaluation of newly derived T scores showed distinguishable patterns between 10058F4 in MYC 390–409 and 10074G5 in MYC 365–384 (Figure 3A), which is in the agreement with the results of previous CD and NMR studies [14]. Besides, RG7112 binding site at MDM2 50–69 showed the highest T score among segments derived from the 50–99 region (Figure 3B). The MDM2 50–69 region contains a α-helix between residues 50 and 64, whereas residues 65–69 form a loop. This region directly contacts RG7112 [21]. The majority of the remaining regions in the 70–90 segments go away from the RG7112. However, a short loop (93–96) comes back to the binding site and contacts RG7112 [21]. Therefore, the already known structural biology study results of the active compound to the intrinsically disordered region are well coordinated with our observations.

This evidence supports our method validity for IDPR binding site inhibition, not limited to direct IDPR inhibition. Another merit of our method is that it does not depend on binding site structure. Although we did not apply our method for fuzzy complex inhibition, we propose that our method is applicable to block such fuzzy complex. NUPR1-MSL1 forms a fuzzy complex, and a chemical compound inhibitor (ZZW-115) was discovered for blocking their complex formation [22,23]. We suggest that our next goal is to validate this compound binding toward the corresponding target regions in NUPR1 [23].

To study binding properties of compounds, we analyzed peptide contacts from molecular docking and compared those contact sites with sites involved in interaction with their target protein structures. To do this from molecular docking result, we evaluated the number of contacts between the peptides and compounds. For double evaluation, we used two contact distance cutoffs (4 Å and 3.6 Å) for contact decision. After mapping those contact residues, we compared the residue positions with the corresponding structures of MYC (PDB ID: 1NKP chain A) in its complex with MAX (Figure 3C). Newly evaluated contact scores from the analyzed docking cases showed significantly higher values than the results of other molecular docking experiments (Figure 3D). 

Our structural comparison study of 10058F4 and MYC confirmed that Y402 serves as an important contact point (Figure 3C, Supplementary Figure S1). According to the previous studies on the 10058F4 and MYC, Y402 was considered as the main contact residue of 10058F4 [11,17,18]. In corresponding analyses, two studies focused on the 402–412 region and used it in the molecular dynamics simulation [17,18]. Because we segmented the 385–424 region, K398 also appeared as the contact residue near Y402. Furthermore, Y402 is embedded within the T400–S405 region between two polar amino acid residues. Therefore, our study suggests that the major MYC-10058F4 binding mechanism is centered at Y402 and the polarity of its environment.

Although they were less frequent than 10058F4–MYC interactions, we also found contacts between ABA and the MYC A401–Y402 region (Figure 3C, Supplementary Figure S1). In our previous study, we applied the Similarity Ensemble Approach (SEA) database to search for the off-targets [24]. Our novel method showed distinguishable contacts for 10058F4, 10074G5, and RG7112 inside their known binding regions as well as the direct/indirect contacts in the disorder-to-order transition region (Figure 3A,B). Although SEA was focusing on structural data, our new method considers diverse forms of disorder-to-order transition regions. MYC A401–Y402 contacts of ABA may provide an evidence of MYC inhibition, which constitutes a new discovery.

### 2.3. Chemical Compound Library Docking to MYC 390–409 Shows Better 10058F4 Scores

Generally, molecular docking aims to find hits from the chemical compound libraries, and this is the reason why the procedure is called “virtual screening”. Instead of virtual screening, we studied the statistical significance of 10058F4 docking through comparison with the results of docking of the members of a valid chemical compound library (Diversity set V of National Cancer Institute). Besides, because of utilization of our new evaluation score function characteristics (Section 3 Materials and Methods), we hypothesized that there is a positive correlation between the docking-involved atom number and a new evaluation score. From the compounds (10058F4, ABA, and APC) and MYC peptide dockings, we discovered that the 10 best results of the MYC 390–409 docking contain only 10058F4 (Figure 2C). Therefore, we applied molecular docking focused on the MYC 390–409 peptide as described in the Section 3 Materials and Methods.

**Figure 4:**
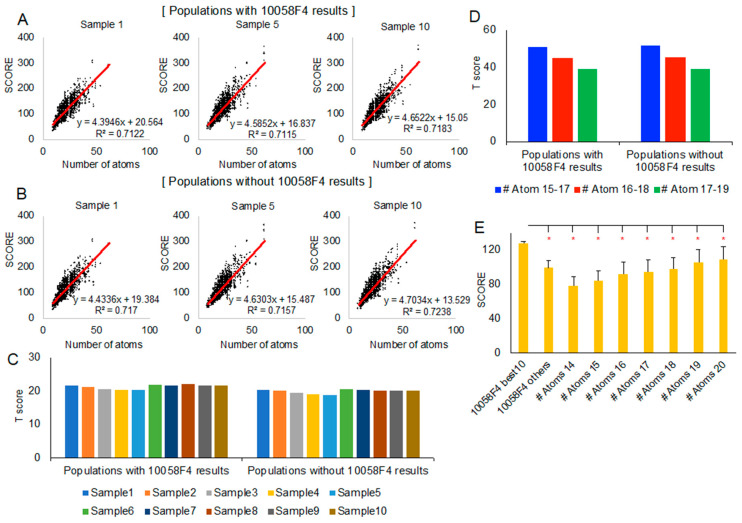
Statistical analysis of the new docking evaluation score focusing on 10058F4–MYC 390–409 region peptides: (**A**,**B**) Linear regression between docking-involved atom number (*x*-axis) and the new docking evaluation score (*y*-axis) with 10058F4 docking results in the population (**A**) and without 10058F4 docking results in the population (**B**). (**C**) The 10 best 10058F4 docking result T scores in 10 random samples (considering each sample as a population) with/without 10058F4 docking results. (**D**) The 10 best 10058F4 docking result T scores in three different docking-involved atom number groups (15–17, 16–18, and 17–19) with/without 10058 docking results from all 10 random samples. (**E**) *T*-test between the 10 best 10058F4 docking results and each of the docking-involved atom number groups (14–20) from all 10 random samples. * *p*-value < 0.001.

To check the correlation between the docking-involved atom numbers and the new evaluation scores, we generated linear regression equation from each population (sample 1, sample 5, and sample 10) (Figure 4A,B). The 22 models of MYC 390–409 peptide docking with 10058F4 (220 results per sample) or without 10058F4 did not show meaningful differences in linear regression. T scores of the 10 best 10058F4 molecular docking results from 22 models, considering each sample as population, were like each other among different populations (Figure 4C). The other remaining 10058F4 docking results (210 results) did not cause significant differences in the T score distribution (Figure 4C).

Because of a new docking evaluation score function with docking-involved atom number normalization, we also grouped all samples (sample 1–10) according to each compound docking-involved atom number. Since the 10058F4 docking-involved atom number was 17, we selected three groups, 15–17, 16–18, and 17–19, for the analysis. As shown in Figure 4D, the population of the 10058F4 docking results did not cause differences in trend. However, the grouped docking-involved atom number increase caused a decrease in the T score of the 10 best 10058F4 docking results. This means that the significance of the score of the 10 best 10058F4 docking results is reduced along the docking-involved atom number increase.

Nevertheless, the 10 best 10058F4 docking results which had many contacts with Y402 (Figure 3C, Supplementary Figure S1) as well as the majority of binding with MYC 390–409 (Figure 2C and Figure 3A) showed greater new evaluation scores than each of the docking-involved atom number 14–20 group average scores (Figure 4E). In the study describing the 10058F4 discovery, the compound was found using the yeast two-hybrid assay, applying 10,000 drug-like small molecules [10]. The following study revealed that the specific 10058F4 binding region in MYC is the 402–412 region [11]. We showed that the 10 best docking results of 10058F4 toward MYC 390–409 have higher scores than other compounds with similar docking-involved atom numbers. Although the binding mechanism between 10058F4 and MYC 402–412 is unclear, we confirmed the previous discovery of specific binding of 10058F4 to Y402 [11,17,18], applying the original definition of the disorder-to-order transition.

### 2.4. ABA and APC Both Have Electrostatic Interaction with MBD2, but Only ABA Shows Specific Electrostatic Interaction with p66α

**Figure 5:**
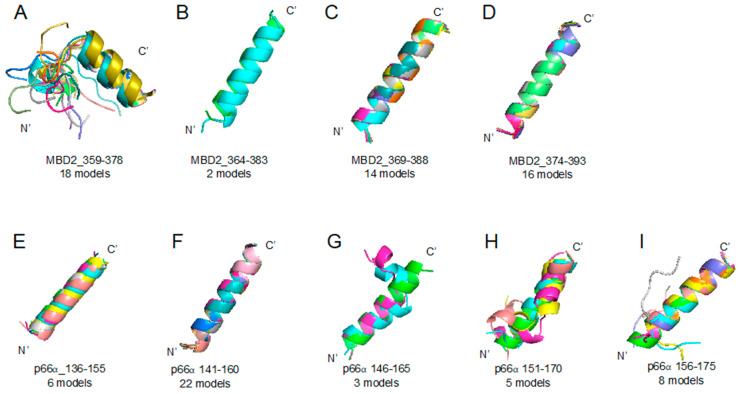
Methyl-CpG-binding domain protein 2 (MBD2) 359–393 region (**A**–**D**) and p66α 136–175 region (**E**–**I**) segmented peptides: PEPFOLD ver 3.5 prediction result of peptide structures based on corresponding sequences (please look at the Table 1). (A) MBD2_359–378 (18 models), (B) MBD2_364–383 (2 models), (C) MBD2_369–388 (14 models), (D) MBD2_374–393 (16 models), (E) p66α_136–155 (6 models), (F) p66α_141–160 (22 models), (G) p66α_146–165 (3 models), (H) p66α_151–170 (5 models), (I) p66α_156–175 (8 models).

We studied ABA and APC interactions with MBD2 and p66α, applying the same procedure used in the 10058F4 analysis. Although we discovered these two compounds from molecular docking to MBD2 and both compounds were shown to efficiently block the MBD2–p66α complex formation [12], it was unclear if there is a binding difference between those proteins. To see their difference using our novel method, the MBD2–p66α (PDB ID 2L2L) [15] complex structure was referred to determine the ABA and APC binding region as a disorder-to-order transition region. Then, we segmented MBD2 (residues 359–393) and p66α (residues 136–175) into the sets of the overlapping 20-amino-acid-long sequences (Table 1) and obtained diverse structures of each peptide region. From diverse forms of peptides (Figure 5), we applied molecular docking and analyzed the result as described in the Materials and Methods section.

**Figure 6:**
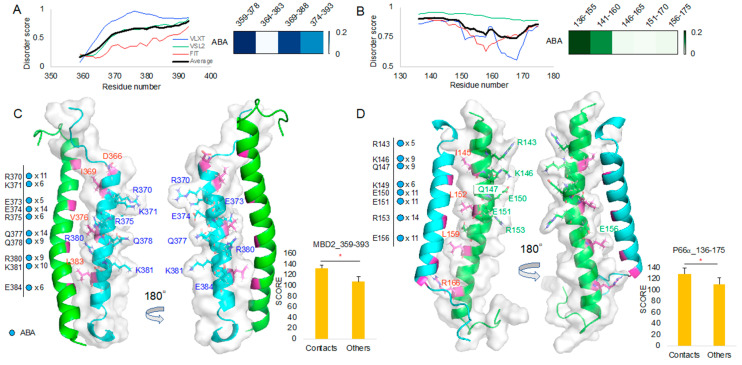
ABA molecular docking toward MBD2 and p66α: (**A**,**B**) Protein intrinsic disorder score plot and the 10 best target region segment peptides ABA docking result T score heatmap of MBD2 (A) and p66α (B). (**C**) MBD2 (cyan) contact residues shown on the MBD2–p66α complex (PDB ID 2L2L) [15] and the corresponding new docking evaluation score (*y*-axis) T-test between contact cases and others. (**D**) p66α (green) contact residues shown on the MBD2–p66⍺ complex (PDB ID 2L2L) [15] and the corresponding new docking evaluation score (*y*-axis) *T*-test between contact cases and others. * *p*-value < 0.001.

ABA appears to have high T score for regions 359–378/369–388 in MBD2 and for the 136–155 region in p66α (Figure 6A,B). Protein intrinsic disorder score patterns did not show meaningful correlation with the T scores as it was in the 10058F4 result (Figure 3A,B). ABA contact mapping revealed residues from the MBD2 backside. These mainly include charged and polar residues, such as R (Arg, +1), K (Lys, +1), E (Glu, −1), and Q (Gln) (Figure 6C, Supplementary Figure S2). These results suggested that electrostatic interactions constitute a main binding mechanism that defines the MBD2–ABA interaction. The p66α structural comparison with the contact mapping also revealed the presence of charged and polar residues, R(Arg, +1), K(Lys, +1), E(Glu, −1), and Q (Gln) from behind the MBD2 binding site (Figure 6D, Supplementary Figure S3). There was no overlap with MBD2–p66α binding site residues [15]. Although we discovered ABA and APC from targeting p66-binding residues of MBD2 [12], the majority of contacts is located behind the binding site. It suggests that ABA interacts with MBD2 behind the p66α binding site and that this interaction prevents MBD2 from p66α binding, likely by inducing the formation of a conformation incapable of MBD2 binding. In p66α as well, ABA binds p66α behind its MBD2-binding site. Therefore, it may also cause the structural changes of p66α, which do not allow MBD2 binding.

**Figure 7:**
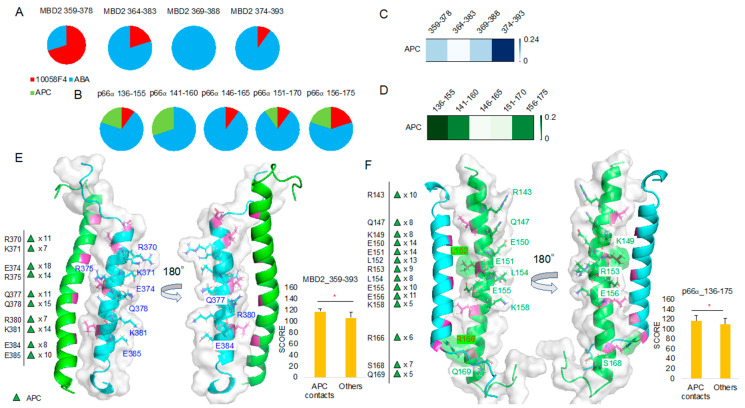
Molecular docking toward MBD2 and p66α, and APC focused docking result analysis: (**A**,**B**) 10058F4, ABA, and APC molecular docking toward MBD2 (A) and p66α (B) and the corresponding 10 best result distribution pie charts. (**C**,**D**) APC-focused docking result T score heatmap from MBD2 docking (C) and p66α docking (D). (**E**) MBD2 (cyan) contact residues shown on the MBD2–p66α complex (PDB ID 2L2L) [15] and the corresponding new docking evaluation score (*y*-axis) *t*-test between contact cases and others. (**F**) p66α (green) contact residues shown on the MBD2–p66α complex (PDB ID 2L2L) [15] and the corresponding new docking evaluation score (*y*-axis) *t*-test between contact cases and others. * *p*-value < 0.001.

To compare ABA and APC binding, we further analyzed the 10 best docking results of the MBD2 359–393 region and p66α 136–175 region in comparison with 10058F4. It turned out that ABA occupies the majority of the peptides derived from the MBD2 364–393 region (Figure 7A) and the p66α 136–175 region (Figure 7B). APC was not ranked among the 10 best docking results for any of the MBD2 359–393 peptides (Figure 7A), but it ranked in the 10 best docking results for the majority of the p66α 136–175 peptides except for the p66α 146–165 peptide (Figure 7B).

Because APC docking scores were lower than those of ABA as shown in Figure 7A,B, we collected only APC docking results and further analyzed them. The heatmap derived using the APC binding T score matrix proved APC preference for the MBD2 374–393 (Figure 7C). However, there was no APC among the 10 best results of the MBD2 374–393 peptide docking. In the case of p66α binding, T score was relatively higher in p66α 136–155 and 141–160 peptides, and APC occupied 25% of the 10 best positions in the p66α 136–155 and 141–160 regions (Figure 7B,D).

MBD2–APC contact mapping (Figure 7E, Supplementary Figure S4) revealed results like the outputs of MBD2–ABA contact mapping (Figure 6C). Contact residues appeared from the MBD2 backside (opposite from p66α-facing side) and were mostly charged and polar ones, R (Arg, +1), K (Lys, +1), E (Glu, −1), and Q (Gln). However, p66α–APC contact mapping (Figure 7F, Supplementary Figure S5) was different from that of p66α–ABA (Figure 6D), and in addition to charged and polar residues R (Arg, +1), K (Lys, +1), E (Glu, −1), and Q (Gln), hydrophobic residue L (Leu) and polar residue S (Ser) were found in the analysis. Despite the fact that no specific interaction mechanisms were identified and no inside–backside distinctions were found, L152 and R166 contact was discovered. Furthermore, the 10 best molecular docking results of 10058F4 and ABA with MBD2 (4 groups of peptides) and p66α (5 groups of peptides) without APC showed greater score than the APC docking results discovered based on the significant contacts of this compound with MBD2 and p66α (Supplementary Figure S6).

## 3. Materials and Methods

### 3.1. Segmented Peptide Preparation, Molecular Docking, and Evaluation Score Update

For mimicking characteristics of intrinsically disordered region, we obtained diverse structures of target disorder-to-order transition region after its segmentation into a set of overlapping 20-amino-acid-long peptides. This length of segmentation was used because a 20-amino-acid-long peptide is optimal for the unfolding and folding studies [25]. We segmented protein sequences into subsets (Table 1) and then uploaded them on PEPFOLD v3.5 web server (https://mobyle.rpbs.univ-paris-diderot.fr/cgi-bin/portal.py#forms::PEP-FOLD3) to predict peptide structures [16]. From the corresponding results (Figure 1 and Figure 5), we obtained representative structures of clusters and applied molecular docking with custom function (smina) of Autodock vina (The Scripps Research Institute, Molecular Biology, La Jolla, CA 92037, USA), targeting the central residue (every peptide tenth residue) Cα coordinates [26,27]. Chemical compound structures in 3-dimensional SDF formats were obtained from the PubChem database (https://pubchem.ncbi.nlm.nih.gov/). Chemical compounds used in this study are 10058F4 (5-[(4-Ethylphenyl)methylene]-2-thioxo-4-thiazolidinone, ZINC12406714, PubChem CID: 1271002), 10074G5 (4-nitro-N-(2-phenylphenyl)-2,1,3-benzoxadiazol-7-amine, ZINC3879010, PubChem CID: 2836600), RG7112 ([(4S,5R)-2-(4-tert-butyl-2-ethoxyphenyl)-4,5-bis(4-chlorophenyl)-4,5-dimethylimidazol-1-yl]-[4-(3-methylsulfonyl propyl)piperazin-1-yl]methanone, ZINC96270381, PubChem CID: 57406853), ABA (2-Amino-N-[[(3S)-2,3-dihydro-1,4-benzodioxin-3-yl]methyl]acetamide, ZINC40430779, PubChem CID: 93602182), and APC ((R)-3-(2-Amino-acetylamino)-pyrrolidine-1-carboxylic acid tert-butyl ester, ZINC60177071, PubChem CID: 66563909). Recent reverse docking study revealed that Autodock vina is biased (shows negative correlation) due to the contact surface area [28]. Therefore, we measured the protein–compound contact surface area [29] and normalized for the number of the molecular docking-involved atoms. We also evaluated the peptide model quality applying DOPE (discrete optimized protein energy) [30] because we obtained structures from the modeling web server. Finally, we designed a new custom score for intrinsically disordered region direct targeting drug discovery evaluation, combining all the aforementioned scores.

$$SCORE=\underset{i<j}{\sum }f_{t_{i}t_{j}\;}\left(r_{ij}\right)\times -k_{B}T\;\ln g^{\left(n\right)}\left(\vec{x_{1}},\vec{x_{2}},\;\ldots ,\;\vec{x_{n}}\right)\;/\;\left(\overset{n}{\underset{i=0}{\sum }}CSA_{i}\;÷n\right)$$

where $\underset{i<j}{\sum }f_{t_{i}t_{j}\;}\left(r_{ij}\right)$ is the smina (Autodock vina) score function [28], $-k_{B}T\;\ln g^{\left(n\right)}\left(\vec{x_{1}},\vec{x_{2}},\;\ldots ,\;\vec{x_{n}}\right)$ is the DOPE score function [30], and $\overset{n}{\underset{i=0}{\sum }}CSA_{i}$ is protein–compound contact surface area calculation algorithm (dr_sasa) score [29]. Normalization of the dr_sasa score with the molecular docking-involved atom number (*n*) was empirical adjustment to calculate atomic contribution.

### 3.2. MYC, MDM2, MBD2, and p66α T Score Heatmap

The new function score was employed for molecular docking evaluation. Each of the 10 best scores of the compound–peptide pair was collected, and we considered each compound docking score from all peptide dockings as a population. Then, we obtained T scores of each compound–peptide pair for the 10 best scores (the 10 best results for each compound) according to the equation below.

(1) $$T=\frac{\bar{x}-\mu }{S\;/\;\sqrt{n}}$$

where $\bar{x}$ is each compound’s 10 best docking results mean score, μ is the population mean score, S is the standard deviation of each compound’s 10 best docking results, and $\sqrt{n}$ is the square root of the number of best 10 docking results ($\sqrt{10}$). After normalization of each compound T score divided by the corresponding compound of all peptide docking T scores sum, we generated heatmaps using Python 3.7 (Python Software Foundation, Wilmington, DE 19801, USA) and matplotlib 3.2 (Plotly, Montreal, QC H2T 2A3, Canada).

### 3.3. MYC, MBD2, and p66α Docking Contact Residue Mapping

Each peptide’s 10 best docking results from MYC 385–424 (5 peptide groups), MBD2 359–393 (4 peptide groups), and p66α 136–175 (5 peptide groups) were collected. Next, we analyzed the contacts between the peptide residue atoms and the compound docking-involved atoms. We applied two distance cutoffs (4 Å and 3.6 Å) for double evaluation (Supplementary Figures S1–S5). If the atom pair exists, we determined it the contact of the corresponding residue. After locating all residues from highest rank (1) to lowest rank (10), bottom-up order, we counted all contacts at a distance ≤4 Å from segmented peptides and summed up the counts. If the summed-up count of a residue was ≥5, the corresponding residue was chosen. The chosen residue contact count sum were placed along the protein target region and compared with the X-ray crystallography determined structure of MYC–MAX (PDB ID 1NKP) [13] and with the NMR structure of MBD2–p66α (PDB ID 2L2L) [15].

### 3.4. MYC 390–409 Peptide Molecular Docking with 10058F4 and Chemical Compound Library (NCI Diversity Set V)

We utilized National Cancer Institute chemical compound library Diversity set V (https://dtp.cancer.gov/organization/dscb/obtaining/available_plates.htm) for molecular docking toward MYC 390–409 peptide 22 models (Figure 1I) to compare with 10058F4. It generated about 350,000 docking results, and we obtained 10 groups of each 3000 docking results (sample size) from random sampling and, then, considered each group as a population for the statistic (T score) calculation based on the new docking evaluation scores.

To see the new score docking-involved atom number correlation, we employed linear regression toward three random samples (sample 1, sample 5, and sample 10) using Microsoft Excel 16.37. For T score calculation, we collected the 10 best 10058F4 docking scores from each random sample (1–10), and considered each random sample with/without 10085F4 docking results as the population. Docking-involved atom number groupings into 15–17, 16–18, and 17–19 were pursued, and we collected all docking results from 10 random samples. The 10058F4 docking-involved atom number is 17; thus, we selected the group range in this manner. In this case, each group with/without 10085F4 docking results was considered as a population. Then, we obtained the T scores of the 10 best 10058F4 docking results according to the equation below.

(2) $$T=\frac{\bar{x}-\mu }{S/\sqrt{n}}$$

where $\bar{x}$ is the 10 best 10058F4 docking results mean score, μ is the population mean score, S is the standard deviation of the 10 best 10058F4 docking results, and $\sqrt{n}$ is the square root of the number of 10 best 10058F4 docking results ($\sqrt{10}$).

Finally, we compared the 10 best 10058F4 docking results with docking-involved atom number groups (14, 15, 16, 17 without 10058F4 results, 18, 19, and 20) using *t*-test. For the analysis, again, we collected docking results according to the docking-involved atom number from all of random samples (sample 1–10).

## 4. Conclusions

The target binding region segmentation generated sets of the overlapping 20-amino-acid-long peptides, and diverse structural forms of these peptides were predicted. We used this step of structural diversity mimicking to study the disordered binding sites that undergo binding-induced folding (i.e., the disorder-to-order transition regions). Molecular docking to these peptides and the new evaluation score successfully distinguished already known compounds and their specific binding sites. Furthermore, we discovered that 10058F4 binds specifically to MYC Y402 and polar residues near this key residue. These observations suggest that the MYC–10058F4 interaction is mainly driven by polarity. Statistically, the new docking score is correlated with the number of contact atoms due to the normalization inside the new docking evaluation score function. Nevertheless, 10058F4 is distinguished rather well from compounds with similar number of atoms randomly chosen from a chemical compound library, NCI Diversity set V. We applied the same procedure for the analysis of other inhibitors targeting disorder-to-order transition regions, ABA and APC. ABA showed contacts with charged residues of MBD2 and p66α located behind each protein partner-binding site. APC also showed contacts with charged residues behind the p66α-binding site of MBD2, but no specific binding mechanism was discovered from the analysis of the p66α docking. In summary, we mimicked structural heterogeneity of the disorder-to-order transition regions based on their native characteristics and evaluated compound binding status with a new score function. We suggest this method as a novel protein intrinsic disorder utilizing a drug discovery platform and hope that it will expand the field of the computer-aided drug discovery.

## Figures and Tables

**Table 1:** Protein target regions and segmented peptides.

Protein	Target Region	Peptides	Sequence (N’–C’)
MYC	350–439	MYC_350–369	TEENVKRRTHNVLERQRRNE
		MYC_355–374	KRRTHNVLERQRRNELKRSF
		MYC_360–379	NVLERQRRNELKRSFFALRD
		MYC_365–384	QRRNELKRSFFALRDQIPEL
		MYC_370–389	LKRSFFALRDQIPELENNEK
		MYC_375–394	FALRDQIPELENNEKAPKVV
		MYC_380–399	QIPELENNEKAPKVVILKKA
		MYC_385–404	ENNEKAPKVVILKKATAYIL
		MYC_390–409	APKVVILKKATAYILSVQAE
		MYC_395–414	ILKKATAYILSVQAEEQKLI
		MYC_400–419	TAYILSVQAEEQKLISEEDL
		MYC_405–424	SVQAEEQKLISEEDLLRKRR
		MYC_410–429	EQKLISEEDLLRKRREQLKH
		MYC_415–434	SEEDLLRKRREQLKHKLEQL
		MYC_420–439	LRKRREQLKHKLEQLRNSCA
MBD2	359–393	MBD2_359–378	CKAFIVTDEDIRKQEERVQQ
		MBD2_364–383	VTDEDIRKQEERVQQVRKKL
		MBD2_369–388	IRKQEERVQQVRKKLEEALM
		MBD2_374–393	ERVQQVRKKLEEALMADILS
p66α	136–175	p66α_136–155	SSPEERERMIKQLKEELRLE
		p66α_141–160	RERMIKQLKEELRLEEAKLV
		p66α_146–165	KQLKEELRLEEAKLVLLKKL
		p66α_151–170	ELRLEEAKLVLLKKLRQSQI
		p66α_156–175	EAKLVLLKKLRQSQIQKEAT

## Supplementary Materials

Supplementary materials can be found at https://www.mdpi.com/1422-0067/21/15/5248/s1. Figure S1. MYC binding region peptide docking result contact mapping, Figure S2. MBD2 binding region peptide docking result contact mapping, Figure S3. p66α binding region peptide docking result contact mapping, Figure S4. APC-focused MBD2 binding region peptide docking result contact mapping, Figure S5. APC-focused p66α binding region peptide docking result contact mapping, Figure S6. APC-focused docking score comparison between APC contact residue mapping result and the other 10 best peptide docking results (MBD2, 4 peptides; p66α, 5 peptides) without APC (wo APC).

## Abbreviations

10058F4	10058-F4: 5-[(4-Ethylphenyl)methylene]-2-thioxo-4-thiazolidinone, ZINC12406714, PubChem CID: 1271002
10074G5	10074-G5: 4-nitro-N-(2-phenylphenyl)-2,1,3-benzoxadiazol-7-amine, ZINC3879010, PubChem CID: 2836600
ABA	2-Amino-N-[[(3S)-2,3-dihydro-1,4-benzodioxin-3-yl]methyl]acetamide, ZINC40430779, PubChem CID: 93602182
APC	(R)-3-(2-Amino-acetylamino)-pyrrolidine-1-carboxylic acid tert-butyl ester, ZINC60177071, PubChem CID: 66563909
CD	Circular dichroism
CSA	Contact surface area
DOPE	Discrete optimized protein energy
DOT	Disorder-to-order transition
EM	Electron microscopy
IDP	Intrinsically disordered protein
IDPR	Intrinsically disordered protein region
MAX	Protein Max
MBD2	Methyl-CpG-binding domain protein 2
MDM2	E3 ubiquitin-protein ligase Mdm2
MoRF	Molecular recognition feature
MYC	Myc proto-oncogene protein
NCI	National Cancer Institute
NMR	Nuclear magnetic resonance
p66α	Transcriptional repressor p66-alpha
RG7112	RG-7112: [(4S,5R)-2-(4-tert-butyl-2-ethoxyphenyl)-4,5-bis(4-chlorophenyl)-4,5-dimethylimidazol-1-yl]-[4- (3-methylsulfonyl propyl)piperazin-1-yl]methanone, ZINC96270381, PubChem CID: 57406853
