# Supplementary material for: Drug Discovery Targeting the Disorder-To-Order Transition Regions through the Conformational Diversity Mimicking and Statistical Analysis
Source: Int J Mol Sci. 2020 Jul 24;21(15):5248. doi: 10.3390/ijms21155248 (PMC7432763; doi:10.3390/ijms21155248)
Supplement: Supplementary file 1 [file ijms-21-05248-s001.pdf]

International Journal of
Molecular Sciences

MDPI

Supplementary Materials

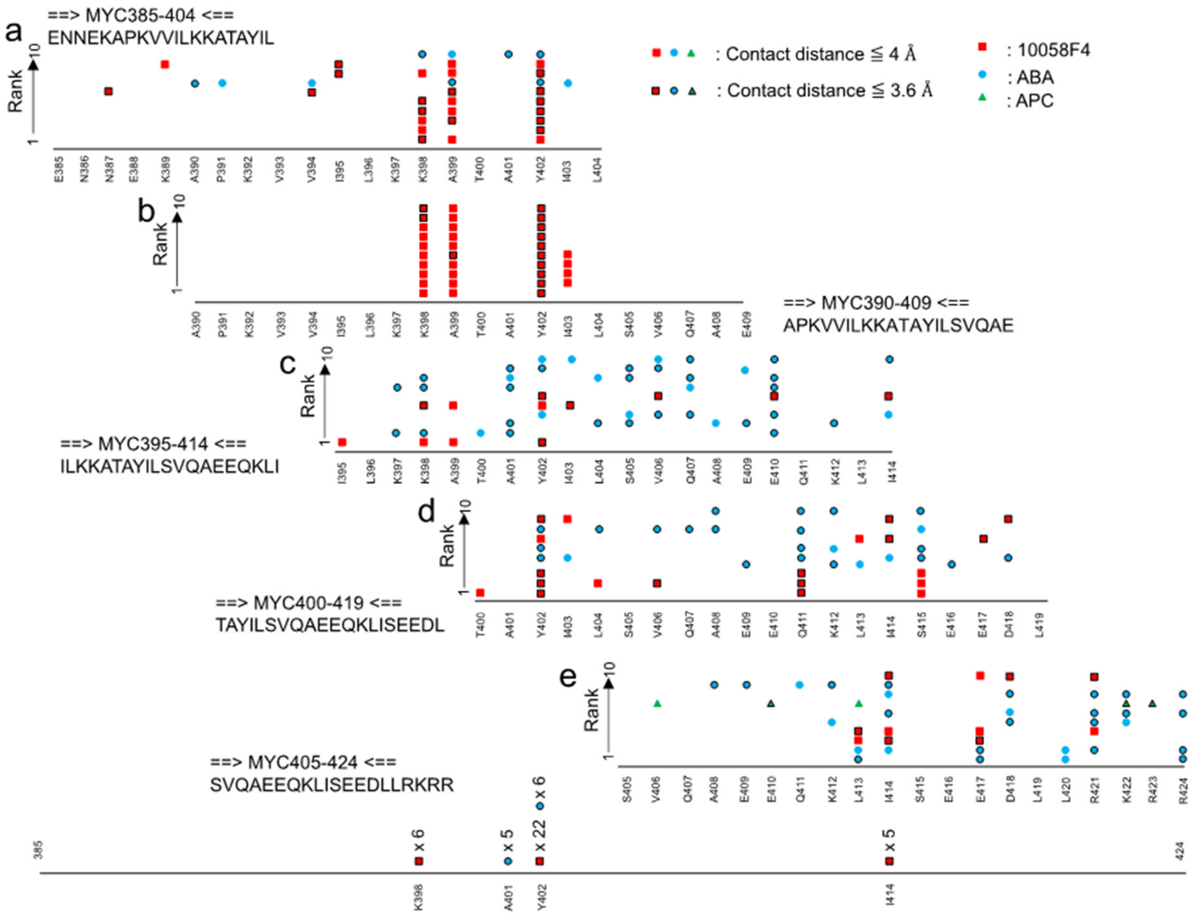

Figure S1. MYC binding region peptide docking results contact mapping. This figure supplements Figure 3C.

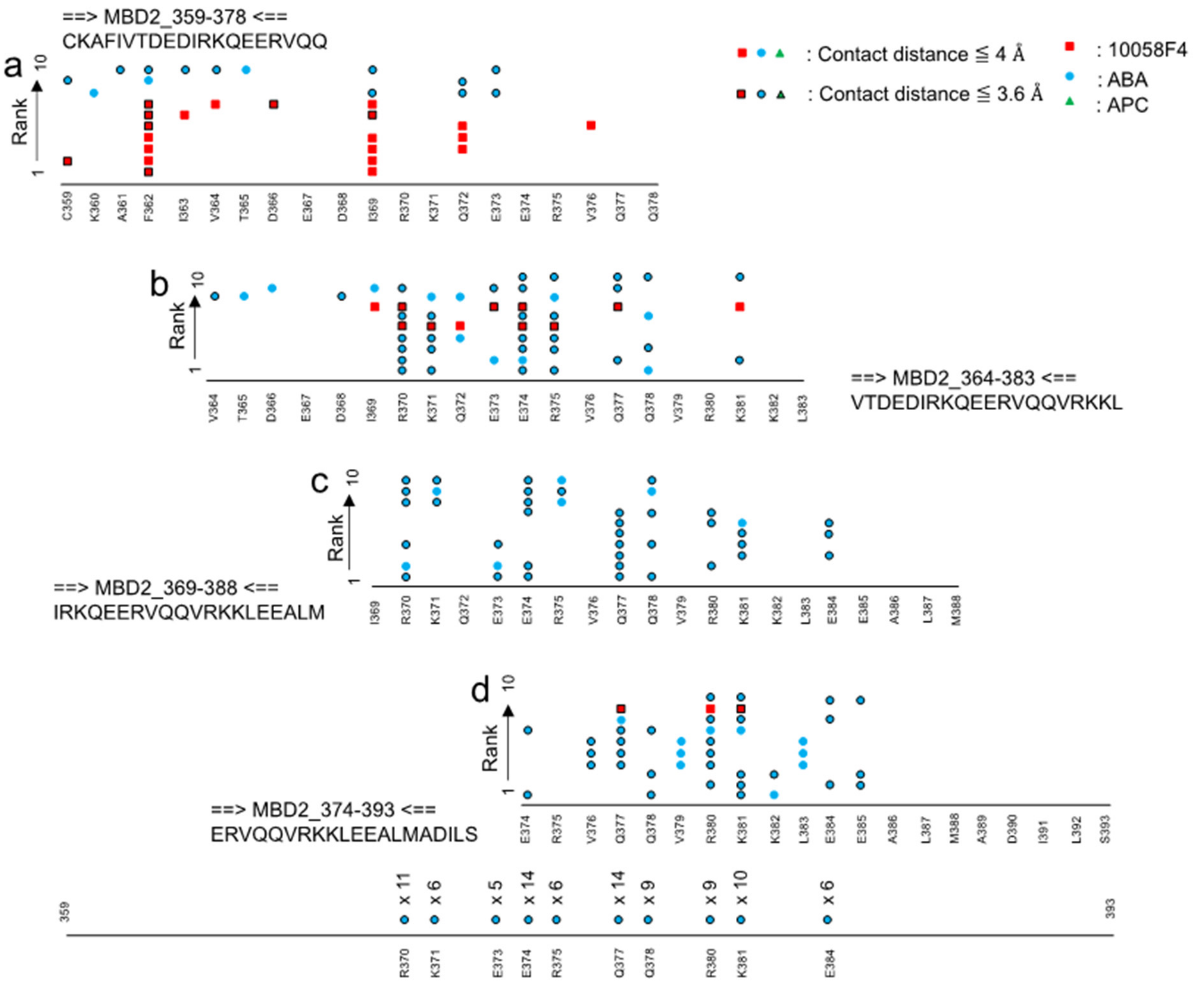

Figure S2. MBD2 binding region peptide docking results contact mapping. This figure supplements Figure 6C.

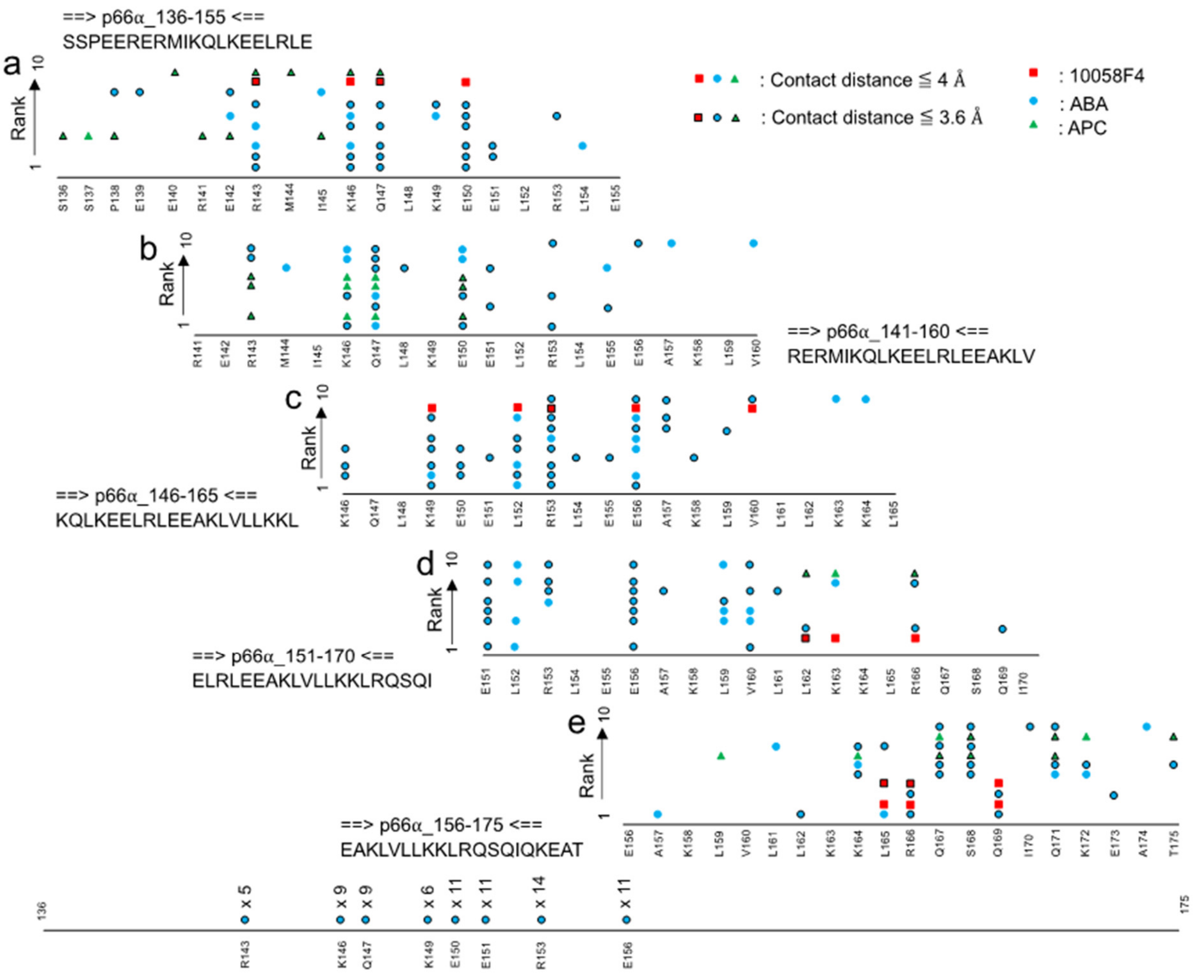

Figure S3. p66α binding region peptide docking results contact mapping. This figure supplements Figure 6D.

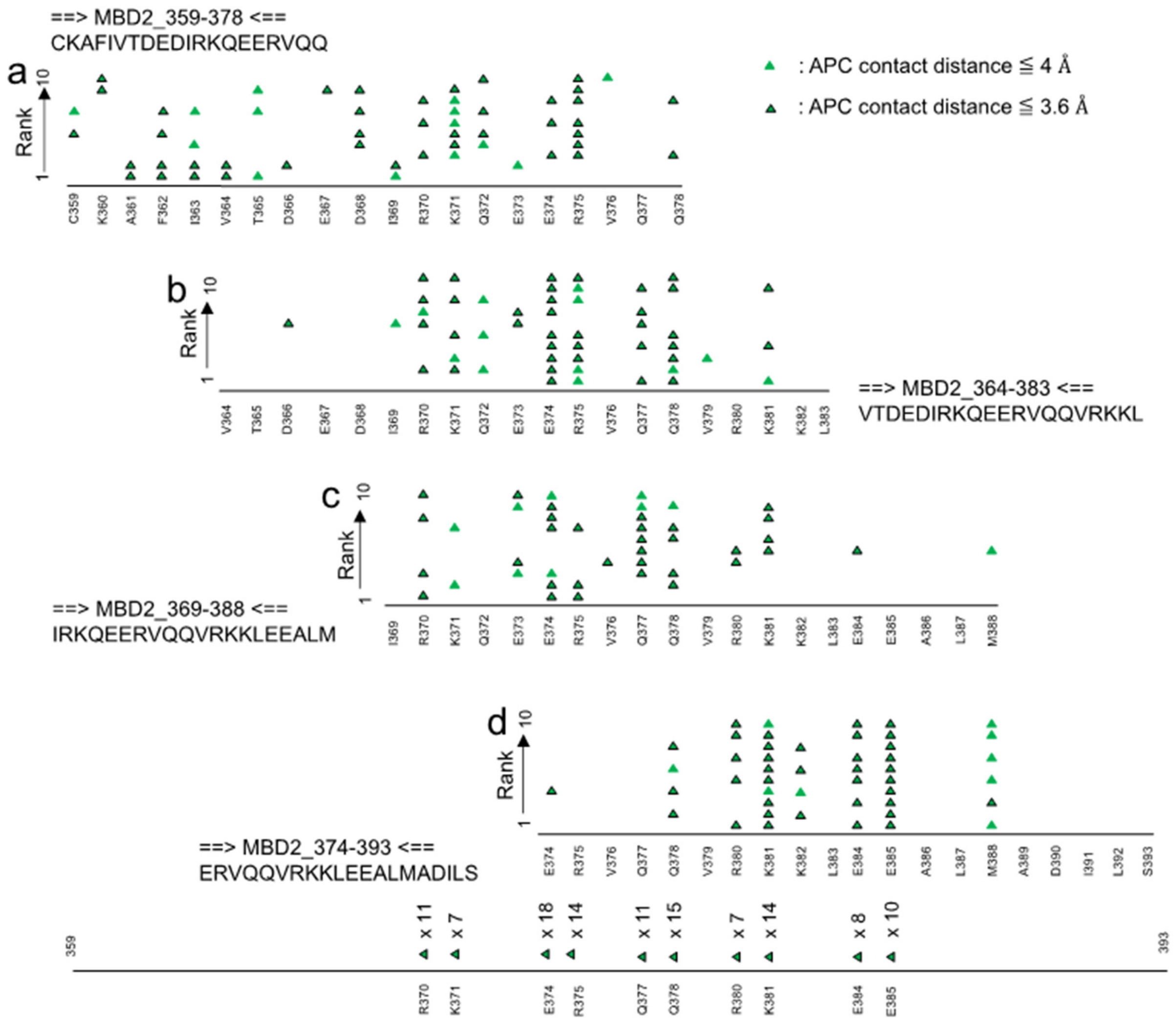

Figure S4. APC-focused MBD2 binding region peptide docking results contact mapping. This figure supplements Figure 7E.

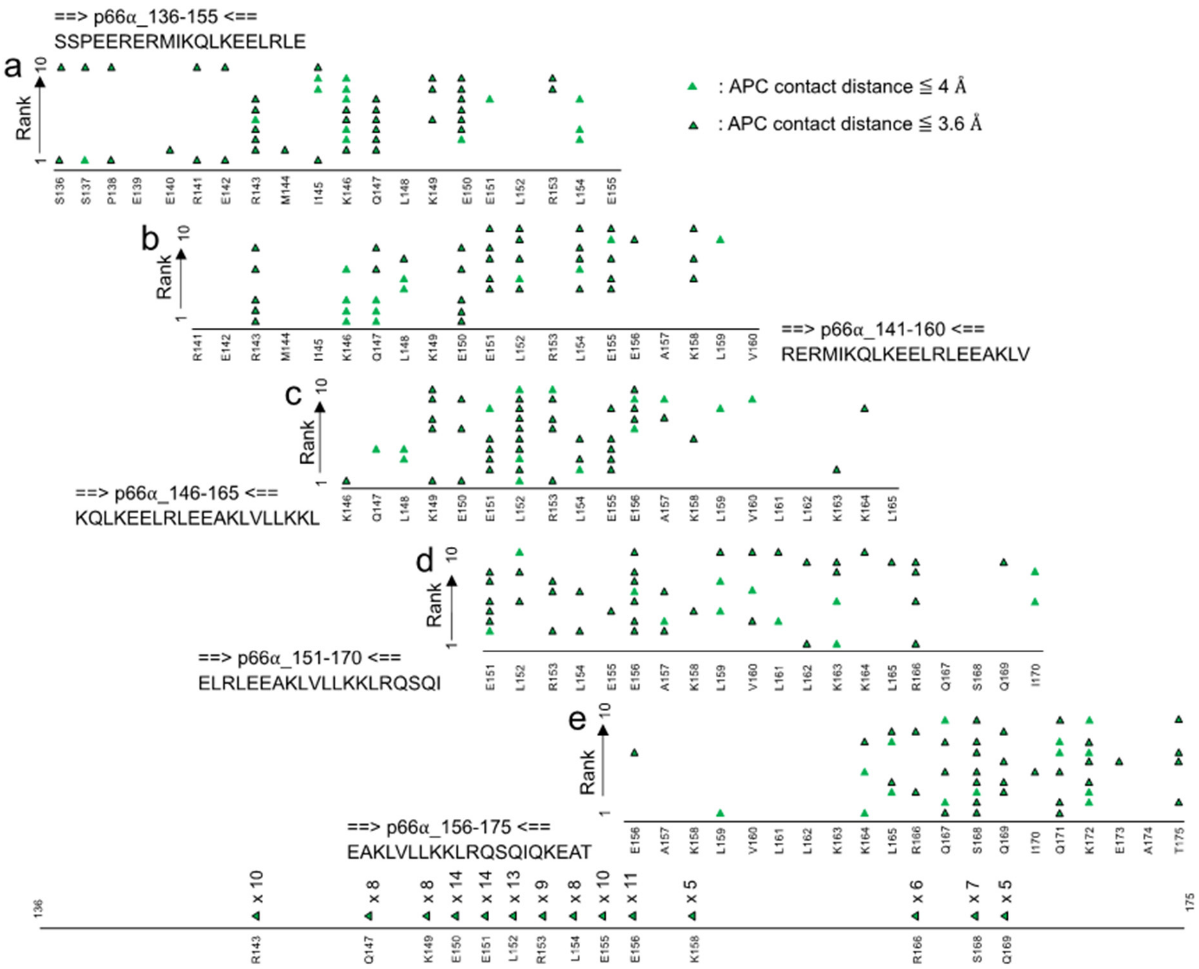

Figure S5. APC-focused p66α binding region peptide docking results contact mapping. This figure supplements Figure 7F.

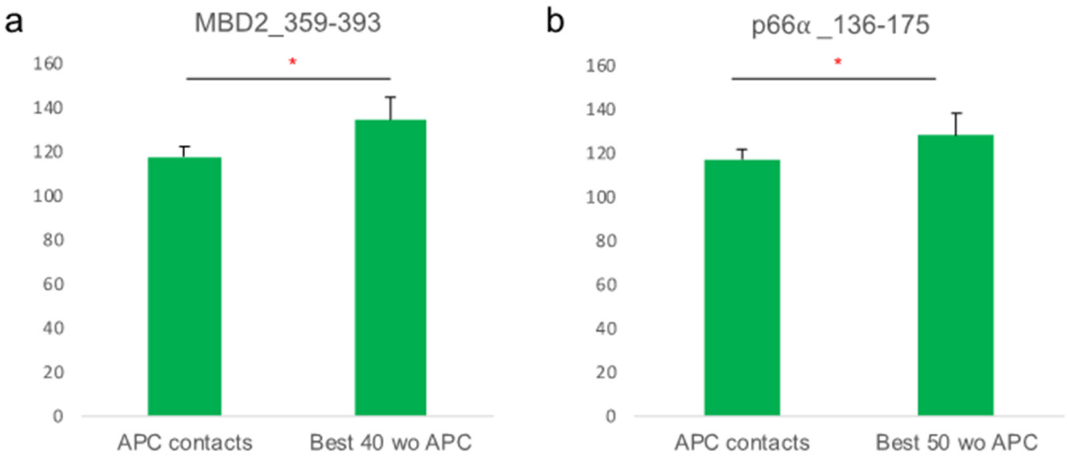

Figure S6. APC-focused docking score comparison between APC contact residue mapping results and other peptide docking best 10 results (MBD2; 4 peptides, p66α; 5 peptides) without APC (wo APC). This figure supplements Figure 7EF. *: p-value < 0.001.
